# Influence of appropriate emergency department utilization and verbal communication on physicians’ (dis)satisfaction with doctor–patient interactions with special consideration of migrational backgrounds

**DOI:** 10.1007/s10354-022-00948-9

**Published:** 2022-07-18

**Authors:** Anna Rahel Pötter, Odile Sauzet, Theda Borde, Baharan Naghavi, Oliver Razum, Jalid Sehouli, Rajan Somasundaram, Hendrike Stein, Matthias David

**Affiliations:** 1 Campus Virchow Clinic, Clinic for Gynecology with Center for Oncological Surgery, Charité University Medicine Berlin, Augustenburger Platz 1, 13353 Berlin, Germany; 2 School of Public Health, Dept. of Epidemiology and International Public Health, Bielefeld University, Bielefeld, Germany; 3 Alice Salomon University of Applied Sciences, Berlin, Germany; 4 Charité Comprehensive Cancer Center, Charité University Medicine Berlin, Berlin, Germany; 5 Department of Emergency Medicine, Campus Benjamin Franklin, Charité University Medicine Berlin, Berlin, Germany; 6 Department of Emergency Medicine, Vivantes Clinic Berlin-Neukölln, Berlin, Germany

**Keywords:** Emergency room, Appropriate utilization, Doctor–patient interaction, Migration experience, Doctor–patient communication, Klinische Notaufnahme, Angemessene Inanspruchnahme, Arzt-Patienten-Interaktion, Migrationserfahrung, Arzt-Patienten-Kommunikation

## Abstract

In recent years, utilization of emergency departments (EDs) has increased continuously, both in Germany and internationally. Inappropriate use of EDs is believed to be partly responsible for this trend. The topic of doctor–patient interaction (DPI) has received little attention in research. However, successful DPI is not only important for adherence and treatment success, but also for the satisfaction of medical staff. This non-interventionl cross-sectional study attempts to identify factors influencing physicians’ satisfaction with DPIs, with a particular focus on the appropriate utilization of EDs and verbal communication. We carried out tripartite data collection in three EDs of major referral hospitals in Berlin between July 2017 and July 2018. Migration experience, communication and language problems, level of education, and a large gap between physicians’ and patients’ perceived urgency regarding the utilization of EDs influence the quality of the doctor–patient relationships and interactions.

## Abstract (de)

In den letzten Jahren hat die Inanspruchnahme von klinischen Notaufnahmen (KNA) sowohl in Deutschland als auch international kontinuierlich zugenommen. Es wird angenommen, dass unangemessene Inanspruchnahmen von KNA mitverantwortlich für diesen Trend sind. Das Thema Arzt-Patient-Interaktion (API) ist in diesem Zusammenhang in der Forschung bisher wenig beachtet worden. Eine erfolgreiche API ist aber nicht nur für die Adhärenz und den Behandlungserfolg wichtig, sondern auch für die Zufriedenheit des medizinischen Personals. Im Rahmen einer nichtinterventionellen Querschnittsstudie wurden Einflussfaktoren auf die Zufriedenheit von Ärzten mit der API identifiziert, mit einem besonderen Fokus auf der angemessenen Nutzung von KNA und der verbalen Kommunikation. Zwischen Juli 2017 und Juli 2018 wurde in 3 großstädtischen KNA in Berlin eine dreigliedrige Datenerhebung durchgeführt. Migrationserfahrung, Kommunikations- und Sprachprobleme, Bildungsniveau und eine erhebliche Diskrepanz bei der wahrgenommenen Dringlichkeit der KNA-Inanspruchnahme beeinflussen die Qualität der Arzt-Patient-Beziehung und der Interaktion.

## Introduction

Emergency departments (EDs) provide immediate medical assistance to seriously ill or injured patients, or patients classified as emergencies by specially trained staff using the resources of a hospital. This includes patient evaluation, stabilization, diagnosis, and indication of subsequent treatment, as well as planning and organization of all further measures. EDs are characterized by their constant availability and a low access threshold. In Germany, the services of EDs can be accessed 24/7 and without any further barriers. In the years prior to the COVID‑19 pandemic, the utilization of EDs has increased steadily at both a national and international level, including the visits by many individuals who had no objective indication for emergency medical treatment [1, 2]. The increased utilization of EDs can lead to overwork and frustration of staff as well as long waiting times for patients due to limited capacity and insufficient structural conditions. In addition, many emergency departments are not adequately equipped to handle the increasing number of patients, neither financially nor in terms of personnel [3].

Health care research studies on the disproportionately high utilization of EDs focus on sociodemographic factors such as gender, age, and migration background (MiB). The utilization behavior of immigrants may differ in type and extent compared to people without migration background or experience. Apparently, these differences can be attributed, among other things, to the individual’s health status, perceived needs, language barriers, sociocultural differences, and traumatic experiences [4].

Medical staff in EDs are particularly exposed to work-related forms of stress due to high treatment needs, time pressure, and the organizational structure of the German healthcare system, in which EDs act as an interface between primary and secondary care.

The diversity of patients’ complaints and needs as well as the actual or perceived urgency of many problems make the job of ED physicians challenging. Patients who visit EDs expect to receive immediate and appropriate care. To date, very few studies have examined physicians’ satisfaction with the doctor–patient relationship in the context of EDs. Babitsch et al. (2008), for example, addressed how ED physicians perceived the relationships between doctor and patient and examined the possible role of the patients’ gender and ethnicity in this [5]. In their review of literature, Cooper et al. (2006) found that the patients’ ethnicity and especially problems associated with communication and language comprehension accounted for differences in the quality of doctor–patient relationships [6]. In their literature review, Ahmed et al. (2017) highlighted that when caring for patients with a migration background, physicians reported more misunderstandings and were more insecure regarding their ability to communicate effectively. As a result, they changed their style of communication and spoke in a more directive way, which may have had a negative impact on joint decision-making [7]. Due to differences in patient populations and patient access to medical resources, it can be assumed that a translation to other countries and patient populations is very limited.

To date there are very few studies from German-speaking countries focusing on medical staff in EDs and their satisfaction with doctor–patient relationships. Thus, the aim of the present study was to address the following questions: how do sociodemographic factors, in particular a migration background, influence the appropriate utilization of EDs? How does the quality of verbal communication influence physicians’ satisfaction with doctor–patient interactions (DPI)?

## Patients and methods

As part of a non-interventional cross-sectional study [8], factors influencing patients’ utilization of hospital emergency departments (EDs) were assessed and analyzed with a focus on how satisfied physicians were with the doctor–patient interactions (DPIs). We collected data from three sources: 1) standardized questionnaire-based interviews with patients, 2) short questionnaires for attending physicians concerning the DPI, 3) evaluation of the patient’s medical report from the ED.

### Patient questionnaires

The guideline for the standardized interviews focused on the following topics (51 items in total): access routes to and expectations of the ED, perception of pain and complaints, self-help measures, social data, questions about general living conditions, and migration/acculturation. The set of questionnaires mainly consisted of predefined response categories. The data were collected by three study nurses, ten student assistants, and one research assistant/project coordinator. To avoid any additional bias, the interviewers were not involved in the medical management of patients.

Our assessment of whether a patient had a migration background or not was based on the patients’ own or their parents’ place of birth. According to the definition of the German Federal Statistical Office during the period of investigation, we distinguished between persons with a migration experience of their own (“population 1”: patients with a first-generation migration background = own migration experience) and their direct descendants (“population 2”: patients with a second-generation migration background = migration experience in the family [at least one parent born abroad, no migration experience of their own]). Patients with no migration background were represented in “population 3”.

All standardized interviews were conducted prior to any contact with physicians but after triage in the ED. The validated questionnaire sets were available in German, English, Turkish, Arabic, and Russian.

### Short structured questionnaire for physicians

The survey among physicians focused on their perceived urgency of treatment as well as their satisfaction with the verbal communication and the DPI. For this purpose, a self-developed short questionnaire was used, which the physicians filled out immediately after they had had contact with the patient. It included the following seven items: 1) assessment of urgency of medical treatment (scale 0 to 10); 2) patient’s native language; 3) language in which the doctor–patient conversation was conducted; 4.a) information on whether an interpreter was appointed; 4.b) details of the person who translated; 5) assessment of language comprehension (scale 1 to 5); 6) assessment of satisfaction with the DPI (scale 1 to 5), with a request for explanation by the doctor using free text if DPI was only assessed as “somewhat satisfying,” “dissatisfying,” or “very dissatisfying”. For reasons of assured data privacy and in order to integrate the survey of physicians into the daily clinical work and routine of the ED, the questionnaire was kept short. Therefore, no sociodemographic data (e.g., age, gender, migration background, professional experience) were collected.

### Satisfaction with the DPI

Physicians’ responses to the question “As a doctor, were you all in all satisfied with the course of the examination/interview?” determined their level of satisfaction with the DPI. Respondents were asked to assess their satisfaction on a five-point Likert scale from 1 (“very satisfied”) to 5 (“very dissatisfied”). For the purpose of statistical analysis, the five response options were combined into two groups (scale values 1 and 2 = “satisfied” with the DPI; scale values 3 to 5 = “dissatisfied” with the DPI).

### Medical reports from the ED

Along with the patient survey, we also collected data from the medical reports of all patients who visited the EDs and were interviewed by the study team. These included administrative data, physicians’ documentation of the medical history and current complaints of the patient, as well as the diagnostic and therapeutic approach. Evaluation of the medical records of the ED was also important in assessing and defining the appropriateness of emergency department utilization. Thus, they recorded the criterion “inpatient admission,” which was used, among other things, to define the appropriateness of ED utilization. All data from the questionnaires and the medical records were first anonymized and then merged.

### Interviewing procedure

The entire data collection took place between July 2017 and July 2018 in two EDs (Charité—University Medicine Berlin, Campus Benjamin Franklin; Vivantes Clinic Neukölln) and one special gynecology ED (Charité—University Medicine Berlin, Campus Virchow Clinic), on all weekdays from 9 am to 11 pm in two shifts. The interviews were conducted by a total of 14 trained interviewers with high competence in relevant foreign languages.

### Exclusion criteria

Patients were excluded from the study if they were younger than 18 years old, if their life was seriously in danger, if they were heavily intoxicated, delirious, or otherwise unresponsive.

### Appropriate utilization of emergency departments

We used four parameters to determine appropriate ED utilization. ED utilization was defined as appropriate if the patient had been admitted as an inpatient (criterion A), or/and if all three of the following criteria were met: 1) patient indicates a treatment urgency of at least 7 out of 10 as well as 2) a pain intensity of at least 7 out of 10, and 3) the decision to seek emergency medical care was made by a physician (criterion B) [8]. The patients were asked to assess their perceived urgency and intensity of pain using an 11-point Likert scale (from 0 = “no urgent need for treatment” to 10 = “very urgent; imminent danger to life”/from 0 = “no pain at all” to 10 = “unbearable pain”).

### Statistics

For the analysis, we employed the data processing software Microsoft Excel (Microsoft Office Excel 2018, Microsoft Corporation, Redmond, WA, USA) and Stata—Statistics Data Analysis (Stata Corp. 2019. Stata Statistical Software: Release 16. College Station, TX: StataCorp LLC.). Following a descriptive analysis of the patient population, we visualized prevalences relating to the quality of DPIs as well as gender- and age-specific differences. Contingency tables were used to examine a possible relationship between physicians’ satisfaction with the DPI and the appropriate utilization of EDs. Then, we employed statistical models for logistic regression as a multivariate method of analysis. After adjusting for possible confounders, we looked for relevant factors influencing the quality of the DPI.

## Results

A total of 4176 patients were invited to participate in the study during the above-mentioned study period. 2339 patients complied (response rate: 56%). Due to missing or unclear information on their possible migration status, 12 patients were excluded later from the study, ultimately allowing the use of data from 2327 patients. The response rate of the corresponding short questionnaires for physicians was 63.3% (i.e., 1473 short questionnaires out of the total of 2327 participating patients). Data from 1356 patients were eventually included in this analysis. We only considered those patients for whom we had both a completed questionnaire according to the standardized guideline interview and the corresponding questionnaire for physicians answering the question about their satisfaction with the DPI.

### Sociodemographic data

Table 1 provides key sociodemographic data for the three study populations. All patients included were between 18 and 98 years old. The study population included more women than men. Due to the multicentric study concept and the fact that the study was conducted at two internal medicine and one gynecology-only ED, the increased proportion of female patients was to be expected. Reasons for using the ED could be divided into 22 different chief complaint categories (e.g., respiratory complaints, vaginal bleeding, psychiatric symptoms). Domestic violence was not represented in these categories. Affected patients are treated exclusively in the surgical ED.

**Table 1:** Sociodemographic data of the three study populations (figures in %)

	Population 1 *n* = 380 (MiB in first generation)	Population 2 *n* = 154 (MiB in second generation)	Population 3 *n* = 821 (no MiB)	Total *n* = 1355
*Gender*				
Female	77.4	86.4	61.9	69.0
Male	22.6	13.6	38.1	31.0
*Level of education*				
Low	23.2	16.2	6.9	18.6
Intermediate	32.0	53.9	60.6	51.9
High	44.8	29.9	22.5	29.6
*Residential proximity to ED*				
Radius < 1 km	28.2	35.8	30.4	30.4
Radius 1–5 km	39.7	37.8	38.8	39.0
Radius > 5 km	32.1	26.4	30.8	30.6

*ED* Emegency department, *MiB* Migration background

Women and men over 64 years of age were the most frequent users of emergency departments. 316 of the patients were of non-German nationality. The nationalities could be assigned to 69 different countries. In total, 28% of the respondents had a migration experience of their own and 11.4% had a family history of migration. 45.6% of the women and 25.5% of the men who took part in the study had a migration background. When stratifying the age of the patients according to migration status, we found that patients without migration background were, on average, significantly older at the time they visited an ED than patients with a first- or second-generation migration background. On average, patients in population 3 were more than 20 years older (median age: 61.3 years, standard deviation 21.0) than patients in population 1 (median age: 41.0 years, standard deviation 15.8), and about 30 years older than patients in population 2 (median age: 31.5 years, standard deviation 11.1) at that time.

### Utilization behavior

Patients in populations 1 and 2 were only half as likely to arrive at the emergency department by ambulance or other means of medical transportation. The time of utilization (during the day vs. evening) was similar in all three populations. Inpatient admissions were significantly more frequent in population 3 (no migration background; Table 2).

**Table 2:** Transportation to the emergency department, differentiated by migration status (figures in %)

	Population 1 *n* = 249 (MiB in first generation)	Population 2 *n* = 103 (MiB in second generation)	Population 3 *n* = 702 (no MiB)	Total *n* = 1054
*Transportation to ED*				
Private	82.7	84.5	59.5	67.5
PTA	1.2	1.0	11.0	7.7
AV/EA	16.1	14.6	29.5	24.9
*Time of utilization*				
During the day (8 am–6 pm)	91.0	89.9	93.5	92.4
Evening (6 pm–11 pm)	9.0	10.1	6.5	7.6
*Inpatient admission*				
Yes	29.2	17.3	52.8	42.3

*AV/EA* ambulance vehicle/emergency ambulance, *ED* Emergency department, *MiB* Migration backgorund, *PTA* patient transport ambulance

### Physicians’ and patients’ perception of urgency

**Fig. 1:**
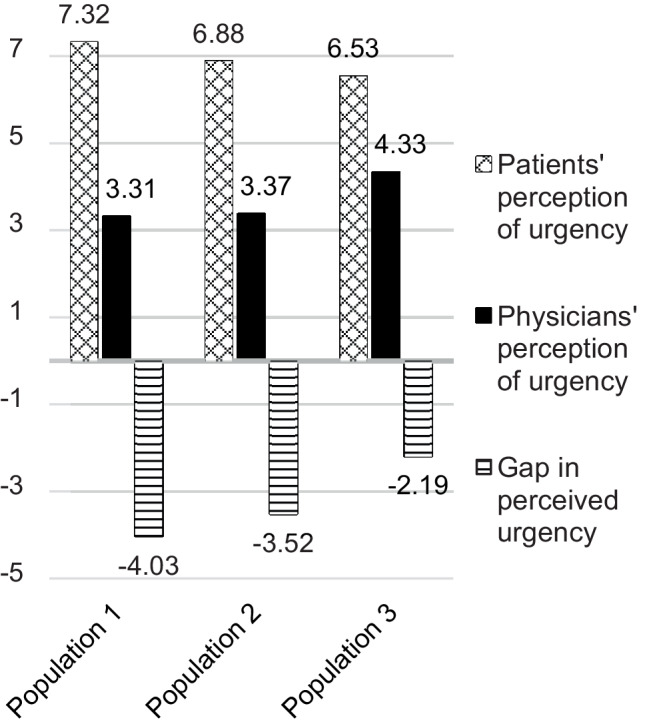
Gap in the perceived urgency of medical treatment (average values for each patient population)

We calculated the difference between physicians’ and patients’ perceptions of urgency. At a value of 0, the perceptions of both parties matched. Negative values indicate that patients considered their complaints to be more urgent than the physicians treating them. Positive values signify that physicians perceived the urgency of medical treatment to be higher than patients. When evaluating the gap in the perceived urgency between patients and physicians, we discovered significant differences. On average, patients considered their complaints to be more urgent than their attending physicians. Population 3 (no migration background) showed the highest level of agreement between the two perceptions, while population 1 exhibited the largest deviation (Fig. 1).

### Index of appropriate utilization

In order to assess the appropriate utilization of EDs, we used the four variables “inpatient admission,” “patient’s perceived urgency of medical treatment,” “patient’s indication of severity of pain,” and “recommendation/decision by registered physicians to visit emergency department.” Following these criteria, every second patient without a migration background made appropriate use of an ED (50%); among first-generation migrant patients this applied to every fourth person (27.1%) and among second-generation migrant patients to every sixth person (16.9%).

### Verbal communication

The majority of doctor–patient conversations with patients from populations 1 (86.9%) and 2 (98.7%) were conducted in German. In only 51 of 1297 patient contacts did the attending physicians use a different language. The most frequently used languages—in descending order—were English, Turkish, French, Arabic, and Polish. Translations of doctor–patient conversations were rare (4.7%) and almost exclusively restricted to conversations with patients who had a first-generation migration background. Translations were mainly provided by persons accompanying the patients (14.6%); professional interpreters were hardly ever used (1.1%).

### Physicians’ satisfaction with verbal communication and DPI

Overall, the attending physicians rated the verbal communication to be “satisfactory” or “very satisfactory” in 88.8% of cases. However, there were considerable differences between the patient groups: population 1: 75.2%; population 2: 96.1%, population 3: 93.1%. Physicians’ satisfaction with DPIs was similarly frequent: population 1: 75.3%; population 2: 92.2%, population 3: 91.9%.

### Predictors for a positive DPI assessment by physicians

#### Model 1

After adjusting for the variables age, gender, education, native language of the patient, and patient’s self-assessed knowledge of the German language, we discovered a significant difference: patients belonging to population 1 (first-generation migration background) were far less likely to receive a positive DPI assessment by physicians compared to patients without a migration background (odds ratio [OR]: 0.44; 95% confidence interval [0.23; 0.83]). Patients who indicated that they had a “good” or “very good” command of German had significantly higher chances that the DPI was considered “satisfactory” by their physician compared to patients who understood “some,” “little,” or “no” German (OR: 1.77; 95% confidence interval [1.02; 3.06]); the quality of the DPI is thus also influenced by language competences. The likelihood of a positive DPI assessment by physicians decreased by a factor of 0.98 (OR: 0.98; 95% confidence interval [0.97; 0.99]) with each additional year of age. A higher education level proved to be a positive predictor: compared to patients with an intermediate-level education, the odds of receiving a positive DPI assessment were higher by a factor of 1.61 (OR: 1.61; 95% confidence interval [1.04; 2.50]) (Table 3).

**Table 3:** Predictors for a positive doctor–patient interaction assessment (logistic regression model 1; *asterisk* *p*-value < 0.05)

	Odds ratio	Standard error	Z	*P*-value	95% Confidence interval	
*Migration status*						
None	1	–	–	–	–	–
First generation	0.44	0.14	2.52	0.012*	0.23	0.83
Second generation	0.83	0.33	0.46	0.644	0.38	1.82
*Native language*						
German	1	–	–	–	–	–
Other	0.45	0.12	2.99	0.003*	0.27	0.76
*Age*	0.98	0.01	3.10	0.002*	0.97	0.99
*Gender*						
Female	1	–	–	–	–	–
Male	0.71	0.13	1.83	0.068	0.48	1.03
*Knowledge of German*						
Some—little—none	1	–	–	–	–	–
Good—very good	1.7	0.50	2.03	0.042*	1.02	3.06
*Level of education*						
Intermediate	1	–	–	–	–	–
Low	0.90	0.20	0.49	0.626	0.58	1.39
High	1.61	0.36	2.13	0.033*	1.04	2.50
*Constant*	19.71	9.01	6.52	0.000	8.05	48.27

#### Model 2

For a second logistic regression model, we replaced the variable “level of education” with the variable “gap in perceived urgency,” which describes the difference between physicians’ and patients’ perceptions of the urgency of medical treatment. After adjusting the variables age, gender, migration background, native language of the patient, and patient’s self-assessed knowledge of German, we discovered a significant difference: patients whose perceived urgency deviated strongly from the physicians’ perception (≥ 3 points more urgent) were less likely to receive a “satisfactory” DPI assessment (OR: 0.54; 95% confidence interval [0.35; 0.83]). As was the case in model 1, the analysis of the other independent variables revealed significant differences for the variables age, native language of the patient, and patient’s self-assessed knowledge of German. In this model, the patients’ migration background had no significant influence on the physician’s satisfaction with the DPI (Table 4).

**Table 4:** Predictors for a positive doctor–patient interaction assessment (logistic regression model 2; *asterisk* *p*-value < 0.05)

	Odds ratio	Standard error	Z	*P*-value	95% Confidence interval	
*Migration status*						
None	1	–	–	–	–	–
First generation	0.52	0.19	1.84	0.065	0.26	1.04
Second generation	0.96	0.43	0.08	0.934	0.40	2.33
*Native language*						
German	1	–	–	–	–	–
Other	0.47	0.13	2.64	0.008*	0.27	0.82
*Age*	0.98	0.01	4.00	0.000*	0.97	0.99
*Gender*						
Female	1	–	–	–	–	–
Male	0.72	0.15	1.61	0.107	0.48	1.08
*Self-assessed knowledge of German language*						
Some—little—none	1	–	–	–	–	–
Good—very good	1.88	0.58	2.04	0.042*	1.02	3.44
*Gap in perceived urgency*						
Low: −3 < x < +3	1	–	–	–	–	–
High: ≤ −3; patients perceive their condition to be more urgent	0.54	0.12	2.81	0.005*	0.35	0.83
High: ≥ +3; physicians perceive the patients’ condition to be more urgent	1.34	0.62	0.62	0.532	0.54	3.33
*Constant*	44.20	22.85	7.33	0.000	16.05	121.74

## Discussion

Medical personnel in EDs are strained by particularly high demands due to high treatment needs, time pressure, and the organizational structure of the German healthcare system, in which EDs act as an interface between primary and secondary care. To date, very few studies have examined how satisfied ED physicians are with the care they provide [9, 10] and other influencing factors. Babitsch et al. (2008) examined how a migration background of patients seeking help affects the satisfaction of ED physicians with the treatment. Their logistic regression analysis showed that the likelihood of physicians being satisfied was significantly lower when they treated patients of Turkish origin than when they treated patients of German origin. The main reasons for their dissatisfaction were communication problems due to patients’ low command of German as well as a perceived lack of urgency for emergency treatment [5]. Our study also demonstrated that most patients perceived their complaints to be more urgent than the ED physicians who treated them. The highest level of agreement was found among patients without migration background. To date, qualitative studies on the reasons patients decide to visit an ED and on their decision-making process are lacking. In order to evaluate whether their visit to the ED is appropriate or not, health-care professionals rely on the perceived severity and urgency of medical problems, while patients focus more on their possibility to access health-care services and on the context in which the medical problem occurred [11].

When a patient’s perceived urgency differed strongly from the physician’s assessment, the ED physicians we interviewed were significantly less likely to consider the doctor–patient interaction “satisfactory” (model 2 of our logistic regression). A native language other than German and a (self-assessed) insufficient knowledge of German also proved to be negative influencing factors. It is a task an ED’s and its medical staff’s task to recognize emergency situations and illnesses that require inpatient treatment or immediate therapy and to initiate or organize diagnostics and treatment. The medical staff are aware that psychosocial factors, relationship and other conflicts, and various nonsomatic causes can also trigger complaints and lead patients to the ED. The observed discrepancy in perceived urgency demonstrates the clash of different “emergency” concepts, which can lead to dissatisfaction.

The “correct” or “appropriate” utilization of EDs is a complex issue. Depending on the definition, the proportion of inappropriate or nonurgent utilization of emergency services in meta-analyses of international studies ranges from 5 to 90% (median 32.1%) [12]. In addition to country-specific influencing factors, this considerable range can be attributed to different definitions of “appropriateness” and dissimilar study populations. Appropriate utilization can be viewed and defined from two different perspectives—from the perspective of providers of health-care services and from the perspective of patients. For our index assessing the appropriate utilization of EDs, we have attempted to combine both perspectives.

The data we collected in detail by means of personal interviews provide information on various characteristics of the study population. This information complements the medical and administrative data summarized in the medical records of the ED.

In this study, we paid special attention to a possible migration background of patients as well as their knowledge and command of German.

In our study, in addition to the three variables “inpatient admission,” “patient’s indication of severity of pain,” and “physician’s recommendation to visit an ED,” we added “patients’ perceived urgency” to our index to assess whether the utilization of the ED was appropriate. According to our index calculations, ED utilization was more often appropriate among patients without a migration background.

Previous studies have also analyzed various characteristics of inappropriate ED utilization, such as general living conditions, psychological and cognitive characteristics, the outpatient medical care situation, and the respective health-related behavior. With regard to outpatient primary care, both poorer local accessibility (“convenience”) and poorer assessment of treatment options of GP practices compared to EDs appeared to be decisive factors [13]. It is important to bear in mind that most patients consider themselves to be in acute need of treatment, which in turn suggests that there is a mismatch between an individual’s health literacy and his or her actual medical indication [14, 15]. In this context, measures to improve patients’ health literacy would be beneficial. Eichler et al. (2009) showed that low health literacy is associated with higher health expenditures per person per year [16]. A study by Zhang et al. (2020) in EDs in Australia found that older people in general, older immigrants, new immigrants, and people without tertiary education or English language skills had a significantly lower health literacy than the general population [17].

The DPI is influenced by a variety of factors. A successful DPI is important for both, treatment success and staff satisfaction. Prolonged feelings of frustration and anger need to be avoided. We found that immigrants of the so-called first generation had significantly lower chances of receiving a positive DPI assessment by physicians than patients without a migration background (model 1 of our logistic regression). Patients who spoke German “well” or “very well” according to their own assessment had a higher chance that their DPI was considered “satisfactory.” On the other hand, non-German-speaking patients were less satisfied with ED services. The provision of a (qualified) interpreter improved the satisfaction of these patients [18]. Language barriers are the main obstacles for good communication with immigrants in EDs, which can negatively affect patients and lead to poor therapeutic compliance, feelings of anxiety, and a desire for a “different” kind of care [19].

### Strengths and weaknesses of the study

This is an interdisciplinary and interprofessional prospective study on a large and representative patient population. Thus, it provides an up-to-date analysis of DPIs in German-speaking countries, taking into account patients with a migration background. The questionnaire set was available in five languages often spoken in EDs. The interview data were merged with both the clinical data of the medical records of the ED and a separate questionnaire for physicians. Limitations were 1) the non-response rate of 44%, including patients who were unwilling or unable to participate due to language barriers (13.6% of all dropouts); 2) the medical record of the ED being a routine medical document and, thus, possibly lacking some data; 3) due to organizational reasons, the daily interviews had to be conducted between 9 am and 11 pm.

## Practical conclusion

- The communication between doctor and patient can potentially be significantly improved by using medically proficient and qualified interpreters as needed. Thus, it is necessary to provide (telephone-based) interpreter services at EDs around the clock.
- It is advisable to improve health literacy and an understanding of the structure of the German health care system among the general population, especially among newly immigrated persons.
- Health care studies must take into account the diversity of patients and apply methods to enable an appropriate representation of different groups. Only with this scientific basis, will we be able to advance patient care in a way that is geared towards both diversity and the future.
